# Association between Berlin Questionnaire Index and lipid profile

**DOI:** 10.5935/1984-0063.20200103

**Published:** 2021

**Authors:** Taina Martins Kikuta, Roberta Souza, Mônica Diniz Rocha Mendel, Juliana Fernandes Batista Pereira, Tharsilla Caríope Azevedo, Ranuzia Mercês Santos Galtieri, Cristina Salles

**Affiliations:** 1 Bahiana’s School of Medicine and Public Health, Medicine - Salvador - Bahia - Brazil.; 2 Bahiana’s School of Medicine and Public Health, Physical therapy - Salvador - Bahia - Brazil.; 3 Federal University of Bahia, Medicine - Salvador - Bahia - Brazil.

**Keywords:** Dyslipidemias, Sleep Apnea Syndromes, Sleep, Sleep Disorders, Circadian Rhythm

## Abstract

**Objective:**

This study aimed to evaluate the association between the Berlin questionnaire index and the lipid profile, according to gender.

**Method:**

This is a cross-sectional study. The group investigated was composed of the Bahiana School of Medicine and Public Health (EBMSP) employees by a sequential non-probabilistic sampling. Study design: The data were obtained by applying a validated questionnaire and collecting laboratory blood samples at the Outpatient Clinics of EBMSP.

**Results:**

The total sample consisted of 94 employees, 21 of whom were excluded because they had not been submitted to blood collection. The sample profile evaluation had a total of 73 employees and obtained the following results in the study: the female was the most prevalent with (54.8%); the age ranged from 18 to 65 years, with a mean 38 ± 10.6 years; in men with positive Berlin (with respiratory sleep disorder), higher values of total cholesterol and LDL-c, and lower HDL-c were observed when compared to men without respiratory sleep disorder, as follows: total cholesterol (202 ± 19 vs. 180±40; p=0.040); LDL-c (137± 17 vs. 113 ± 34; p=0.048); lower HDL-c (37 ± 6 vs. 42,5 ±8; p=0.047). While in women, no significant change was observed among those with positive Berlin when compared with those with negative Berlin.

**Conclusion:**

The findings of this study showed that men with positive Berlin (with a respiratory sleep disorder) had higher values of total cholesterol, LDL-c, and lower HDL-c when compared to men without a respiratory sleep disorder.

## INTRODUCTION

The relationship between sleep restriction and dyslipidemia can be explained by mechanisms regardless of obesity and the developed food preference^1^. A 4-year study following children in their late childhood transition to adolescence found that in girls, the reduction in sleep time and the lower quality of sleep may be related to an increase in cholesterol markers (HDL and LDL). At the same time, in boys, there were more significant associations between sleep and triglycerides. These findings may be related to both the hormonal changes of ghrelin and leptin but may also have an independent relationship^2^.

Such independence was discussed in a cross-sectional study with patients with OSAS, in which there was a positive association between OSAS and increased levels of LDL and triglycerides. The previously held theory was that nocturnal hypoxia explained the increase in these rates. It has also shown that sleep fragmentation plays a significant role in lipid homeostasis. Sleep fragmentation is a stressor that activates the hypothalamic-pituitary-adrenal axis causing elevation of cortisol and adrenocorticotrophic hormone, which performs an essential part in lipolysis and may influence serum lipid levels. Besides, sleep fragmentation causes systemic inflammation, which alters lipid metabolism by redirecting lipoproteins to the lesion site, binding to bacterial products, and consequently protecting the host^3^. Therefore, screening for hypercholesterolemia is relevant, not only in patients with OSAS but also in anyone with fragmented sleep^4-6^.

Based on the above, the present study aims to evaluate the association between the Berlin questionnaire index and the lipid profile in medical school employees, according to gender.

## MATERIAL AND METHODS

This is a cross-sectional study performed at the Bahiana School of Medicine and Public Health. The study was referred to the Ethics Committee of the Bahiana School of Medicine and Public Health and approved under the opinion number 2,333,522 on October 17, 2017. Employees over 18 years of age, when agreeing to take part in the study, signed a free and informed consent term, as determined by Resolution N. 466/12 of the National Health Council.

The following inclusion criteria were used: patients aged 18 years or older; the possibility of answering to the Berlin questionnaire^7^; allowing the evaluation of serum levels of total cholesterol, HDL-c, and triglycerides, after fasting for 8 to 10 hours. As exclusion criteria: patients with diagnosis and treatment for sleep disorders; associated genetic syndromes; neurological diseases; pregnant women.

### Study tools

1. Berlin questionnaire: patients with at least 2 of 3 positive categories were considered at high risk for OSAS. Category 1 (snoring assessment) was positive with two or more positive responses among five questions; category 2 (daytime sleepiness) positive with two or more positive answers among three questions; and category 3 will be positive if the patient is hypertensive or presents BMI>30^7^. The Portuguese version used was validated for Brazilian population^8^.
2. Height: was recorded in the Frankfort plan, with the scale stadiometer with sensitivity up to 0.5cm.
3. Weight: a single examiner carried out the measurements, with participants barefoot and with the least amount of clothing possible, on a scale Filizola. Determinations were made up to the level of 100 grams.
4. BMI: after measures of weight and height, BMI was calculated using the ratio between the individual’s body mass (in kilograms) by the square of his/her height (in meters) and determined in kg/m^2^. Adopting the categories applied by the World Health Organization (WHO): low weight (<18.5), normal weight (18.5-24.9), overweight (25.0-29.9), obese I (30, 0-34.9), obese II (35.0-39.9) and obese III (>40.0).
5. Age: according to the date of birth reported in the protocol, age was measured in full years.
6. Sex: variable obtained through the questionnaire.
7. Race: the breed was considered self-defined according to the official terminology of the Brazilian Institute of Geography and Statistics (IBGE), adopting as reference the color of the skin (white, black, yellow, brown, or indigenous).
8. Blood sample: the lipid profile (total cholesterol, HDL-c, LDL-c, and triglycerides) was evaluated using the spectrophotometry method in automatic equipment BT 300 plus from Wiener Lab. Blood collection was performed in the ADAB laboratory after an 8-10 hour fast. “Dyslipidemias and dyslipidemias prevention” was used for the laboratory classification of dyslipidemias, with a total cholesterol <190mg/dL, desirable HDL-c >40mg/dL, LDL-c <160mg/dL, and triglycerides <150mg/dL.

### Protocol for data collection

Firstly, there was publicity through posters, the EBMSP website, and a face-to-face invitation. Participants became aware of the research objectives and procedure and signed the informed consent form. This data collection was carried out by applying the Berlin questionnaire on the October and November 2017 weekdays. The blood collection was performed after fasting for 8 to 10 hours, in the ADAB laboratory, on the November and December weekdays. The lipid profile (total cholesterol, HDL-c, and triglycerides) was evaluated using the spectrophotometric method in automatic equipment BT 300 plus of the company Wiener Lab.

### Sample calculation

The group studied comprised EBMSP employees, by a non-probabilistic sampling of the sequential type. We used the PEPI-SAMPLE program (Sagebush Press, Salt Lake City, UT, USA) for the sample size calculation, and we adopted the following parameters: confidence level of 90%. The sample was taken from a population of approximately 1,000 employees hired by EBMSP. Therefore, the calculated sample size included 71 employees to fulfill the objectives.

### Statistical analysis

The statistical software SPSS (Statistical Package for the Social Sciences) version 14.0 for Windows was used for tabulation and data analysis. Quantitative variables were expressed as mean ± standard deviation or median and interquartile range (IQR). Qualitative variables were expressed through simple and relative frequencies. We used the Student’s t-test to compare two means. A significance level of *p*<0.05 was considered.

## RESULTS

The total sample consisted of 94 employees of the EBMSP, 21 of whom were excluded because they did not perform the blood collection. The sample profile evaluation had a total of 73 employees and obtained the following results in the study: the most prevalent gender was female with (54.8%); the age ranged from 18 to 65 years, with a median of 37 (29-44) years, and mean 38 ± 10.6 years; the most prevalent ethnic groups were black and brown with a frequency of 93.2% of the investigated population; the mean BMI was 26.4±4.7kg/m^2^, with no significant difference between genders, whereas none of the individuals had grade 3 obesity, and only 15% of them presented some type of obesity, according to the WHO (Table 1).

**Table 1:** Socio-demographic and BMI characteristics of EBMSP employee sample.

Variable	n = 73	%
Gender		
Female	40	54,8
Male	33	45,2
Ethnicity		
White	02	2,7
Black	41	56,2
Yellow	03	4,1
Brown	27	37,0
BMI		
Low weight	04	5,5
Normal weight	24	32,9
Overweight	34	46,6
Obesity I	09	12,3
Obesity II	02	2,7

BMI: Body mass index.

**Figure 1:**
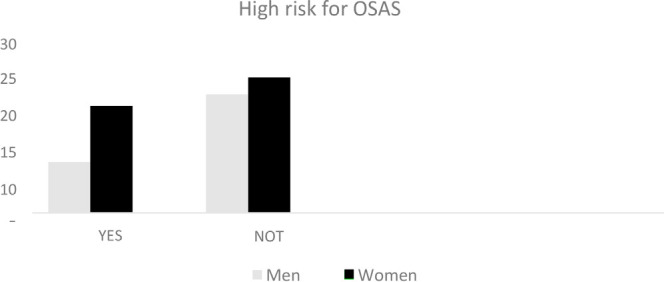
Sex ratio and high risk for OSAS.

Analyzing the results of the Berlin questionnaires, 28 of the 73 study participants presented two or more positive categories of the inquiry. According to this data, 38.4% of the employees studied were at high risk for developing OSAS (obstructive sleep apnea syndrome), 67.85% of whom were women (Figure 1).

When comparing the lipid profile between the genders by Student’s t-test, the mean HDL-c was higher in females than in males (51±12mg/dL vs. 41±8mg/dL, *p*=0.001), while mean triglycerides were higher in males compared to females (125±81mg/dL vs. 88±47mg/dL, *p*=0.23). The mean total cholesterol was similar between males and females (186±37mg/dL vs. 187±37mg/dL, *p*=0.895), and the mean LDL-c was similar between males and females (120±32mg/dL vs. 118±33mg/dL, *p*=0.886). Table 2 shows that there was a statistically significant difference in both patients with a positive Berlin questionnaire and those with negative Berlin concerning HDL-c when comparing female to male (Table 2).

**Table 2:** Comparison of the correlation between the lipid profile and the Berlin questionnaire index between the genders.

Variable (mg/dL)	Berlim positive			Berlim negative		
	Male	Female	p*	Male	Female	p*
Triglycerides	137 ± 47	91,5 ± 39	0,013	121 ± 91	84 ± 53	0,116
Total cholesterol	202 ± 19	188 ± 39	0,217	180 ± 40	186 ± 35	0,593
LDL-c	137 ± 17	119 ± 35	0.740	113 ± 34	118 ± 32	0,612
HDL-c	37 ± 6	51 ± 14	0.010	42,5 ± 8	51 ± 10,5	0,004

LDL-c: low-density lipoprotein; HDL-c: high-density lipoprotein.

* Student T-Test.

When comparing the triglyceride value among individuals with positive Berlin, there was a difference between the genders. In contrast, in subjects with negative Berlin, there was no such difference, with serum triglycerides being higher in the male population (Tables 3 and 4).

**Table 3:** Comparison of the correlation between the lipid profile and the Berlin questionnaire index in male sex.

Variable (mg/dL)	Berlim positive	Berlim negative	p*
Triglycerides	137 ± 47	121 ± 91	0,622
Total cholesterol	202 ± 19	180 ± 40	0,040
LDL-c	137 ± 17	113 ± 34	0,048
HDL-c	37 ± 6	42,5 ± 8	0,047

LDL-c: low-density lipoprotein; HDL-c: high-density lipoprotein.

* Student T-Test.

**Table 4:** Comparison of the correlation between the lipid profile and the Berlin questionnaire index in females.

Variable (mg/dL)	Berlim positive	Berlim negative	p*
Triglycerides	91,5 ± 39	84 ± 53	0,637
Total cholesterol	188 ± 39	186 ± 35	0,843
LDL-c	119 ± 35	118 ± 32	0,919
HDL-c	51 ± 14	51 ± 10,5	0,965

LDL-c: low-density lipoprotein; HDL-c: high-density lipoprotein.

* Student T-Test.

In men with positive Berlin (with a respiratory sleep disorder), higher values of total cholesterol and LDL-c, and lower HDL-c were observed when comparing to men without a respiratory sleep disorder. While in women, we found no significant change between those with positive and negative Berlin.

## DISCUSSION

In the present study, when assessing males at high risk for OSAS investigated through the Berlin questionnaire, we observed higher values of total cholesterol and LDL-c and lower values of HDL-c, when comparing to those men without this risk. The positive relationship between lipid profile and OSAS is also described in the literature, as in the study by Coughlin et al. (2004)^9^, who conducted case-control type research to assess the association between OSAS and metabolic syndrome. To meet this aim, they studied 61 men from the case group, who should have a polysomnographic diagnosis of OSAS, and 43 men from the control group, whose diagnosis of OSAS was discarded. Thus, they concluded that higher triglyceride and lower HDL rates were associated with OSAS. By performing multiple regression, triglycerides lost significance, and HDL emerged as an independent variable for OSAS. Zgierska et al. (2000)^10^ obtained a similar result when studying 73 individuals to assess the association of OSAS with risk for cardiovascular disease. They divided the sample into three subgroups (obese with OSAS, non-obese with OSAS, and obese without OSAS). The authors could conclude that triglycerides maintained a positive and independent association for OSAS after adjusting for obesity; they observed associations between sleep disorder and serum lipids, regardless of obesity, which is not in agreement with the findings in this study.

Opposing these results, Drager et al. (2005)^11^ studied 12 healthy volunteers, 15 patients with mild to moderate OSAS, and 15 patients with severe OSAS; however, they found no statistically significant differences between the groups in terms of OSAS polysomnographic data and lipid profile. Tokuda et al. (2008)^12^ selected 68 men, who were submitted to polysomnography and subsequently divided them into a control group, moderate OSAS group, and severe OSAS group. In this study, they also found no differences between groups regarding serum lipid levels. Although there is no consensus regarding the association between OSAS and lipid profile, sleep fragmentation can act as a stressor factor that activates the hypothalamus-hypophysis-adrenal axis, releasing the hormones cortisol and adrenocorticotrophic, which in high concentrations predispose to lipolysis, hence influencing the serum levels of lipids. Sleep fragmentation in OSAS can produce a systemic inflammatory response, redirecting lipoproteins to the site of the injury for protection, thus increasing the serum levels of these^3-5^.

Contrary to the male sample analysis, the female population at high risk for sleep disorder did not present differences in the lipid profile compared to the female sample without the risk. This result was maintained even adjusting the obesity variable since women presented BMI levels similar to the male population. The fact that estrogen influences the lipid profile as a protector can explain this so that until the fifth decade of life, men tend to have higher mean values, and beyond this age group, women exceed these levels^13^; corroborating with the current survey that presents a sample with a mean age above this delimiting range.

Few studies in the literature aim to screen for sleep disorders using the Berlin questionnaire and its relationship with lipid alterations, as in our research. In this study, we concluded that male individuals at high risk for OSAS, selected using the Berlin questionnaire, had higher levels of total cholesterol, LDL-c, and HDL-c. In agreement, Mattos et al. (2020)^14^ evaluated sleep disorders and their relationship with the lipid profile in 66 individuals with type 1 diabetes mellitus. The Pittsburg sleep quality index, Epworth sleepiness scale, and Berlin questionnaire determined sleep quality. These authors noted that LDL-c and total cholesterol levels were increased in thirty individuals with screened compromised sleep quality.

As a limitation of the study, we did not investigate the following variables: diet, physical activity, and body composition. However, most studies that show an association between lipid profile and OSAS did not consider diet, physical activity, and body composition as confounding factors^15^. As a second limitation, we know the gold standard for diagnosis of OSAS to be polysomnography; however, studies have shown the high reliability of the Berlin questionnaire, through high sensitivity and specificity, allowing comparison of results. Szymanski et al. (2013)^16^ studied 158 patients to assess their risk for OSAS by applying the Berlin questionnaire. The 53 patients, who presented high clinical suspicion for OSAS, by the result of the questionnaire, were subsequently submitted to polysomnography, in which they diagnosed 48 of them for OSAS. Therefore, about 90% of the suspects for OSAS were confirmed, showing the reliability of the questionnaire. Since polysomnography is not always available in health care institutions, the suspicion of obstructive sleep apnea can be stimulated through validated questionnaires with high sensitivity and specificity, allowing the scientific research results to reach the medical protocols.

The present study allows the conclusion that higher values of total cholesterol, LDL-c, and lower HDL-c were observed in men with positive Berlin (with a respiratory sleep disorder) when compared with men without a respiratory sleep disorder. While in women, no significant change was observed among those with positive Berlin compared to those with negative Berlin.
